# Tissue-Derived Biological Particles Restore Cornea Properties in an Enzyme-Mediated Corneal Ectatic Model

**DOI:** 10.3390/bioengineering6040090

**Published:** 2019-09-27

**Authors:** Hongbo Yin, Xiaokun Wang, Shoumyo Majumdar, Jeeyeon Sohn, Byung-Jin Kim, Walter Stark, Jennifer H. Elisseeff

**Affiliations:** 1 Department of Ophthalmology, Sichuan University, Chengdu 610041, China; hongbo_yin@163.com; 2 Wilmer Eye Institute, Department of Ophthalmology, Johns Hopkins School of Medicine, Baltimore, MD 21231, USA; xwang110@jhmi.edu (X.W.); shoumyo.m@gmail.com (S.M.); schwarzbkim@gmail.com (B.-J.K.); wstark@jhmi.edu (W.S.); 3 Translational Tissue Engineering Center, Wilmer Eye Institute and the Department of Biomedical Engineering, Johns Hopkins University, Baltimore, MD 21231, USA; 4 Department of Chemical and Biomolecular Engineering, Johns Hopkins University, Baltimore, MD 21218, USA; jeeyeon518@gmail.com

**Keywords:** extracellular matrix, corneal mechanics, keratoconus, collagen crosslinking

## Abstract

**Purpose:** To investigate the impact of tissue derived biological particles on enzyme-mediated weakened corneas. **Methods:** Rabbit corneas were treated with enzymes to create an ex vivo ectatic model that simulated representative characteristics of keratoconus (KC). Porcine cornea, cartilage, and lymph node tissues were processed to remove most cellular components and cryomilled into microparticles. The KC corneas were cultured in medium containing the tissue-derived biological particles (TDP) overnight. The mechanical, thermal, ultrastructural changes, and gene expressions of corneal stromal cells were characterized to evaluate the effects of the TDP treatment. **Results:** The enzyme treatment significantly reduced corneal mechanics and thermal stability, and also disrupted the extracellular matrix ultrastructure. After culturing with TDP medium, the Young’s modulus of the modeled KC corneas increased by ~50%, comparable to normal cornea controls. Similarly, the thermal denaturation temperature of the corneas was restored. These findings also corresponded to a significant increase in collagen fibril density after TDP treatment. Furthermore, corneas cultured in TDP medium significantly downregulated expression of the pro-inflammatory gene Tnfα, and restored the expression of the key keratocyte markers *Aldh*, *keratocan*, and *biglycan*. **Conclusions:** Tissue-derived biological particles reinforce mechanical and thermal properties of corneal tissue in an ex vivo model of KC. Through this study, we demonstrate and characterize the previously unexplored impact of tissue-derived biological scaffolds on corneal biomechanics, thermal stability, and gene expression, presenting a potential new therapy for ocular disease.

## 1. Introduction

The cornea is the outermost layer of the eye, which functions as a protective and light-refractive tissue. Corneal stroma represents about 90% of the cornea thickness and is composed of a collagen-rich extracellular matrix (ECM) with highly aligned collagen fibrils forming densely packed lamellae [1]. This unique stromal ultrastructure provides transparency and mechanical stability to the cornea. Diseases or injuries of the cornea that cause changes in the matrix ultrastructure are detrimental to corneal mechanical and optical properties and generally cannot be repaired. Keratoconus is an ocular disorder caused by abnormal collagen degeneration, or corneal ectasia, which leads to weakening of the corneas, irregular astigmatism, and vision loss [2]. The ultrastructural damage and disrupted collagen alignment in keratoconic eyes has been previously established [3,4]. Collagen crosslinking initiated with riboflavin and ultraviolet-A (UVA) irradiation is a recently developed therapeutic method designed to slow or halt the progression of keratoconus or post laser-assisted in situ keratomileusis (post-LASIK) ectasia [5]. While corneal crosslinking significantly improves the mechanical characteristics of the cornea [6], exposure to UVA holds some risks. Prolonged UVA irradiation can cause extensive keratocyte loss and even corneal endothelial cell damage [7,8]. Moreover, the treatment does not address the underlying cellular abnormalities. Alternative methods to restore corneal mechanics and homeostasis are needed.

The extracellular matrix (ECM) is composed of proteins and proteoglycans and is the framework in which cells reside in tissues. The ECM contains structural and biological cues that provide instructions to cells that can, in turn, feedback and regulate the matrix. In the case of corneal development and corneal wound healing, proteoglycan ECM components secreted by keratocytes play an important role in regulating corneal fibril size and spacing. This ECM regulation has a critical impact on corneal optical and mechanical properties [9,10]. It also has been reported that ECM proteins such as the decorin core protein can reinforce porcine corneas ex vivo [11]. As part of the natural aging process, proteoglycan ECM components play a role in corneal crosslinking via the Advanced Maillard Reaction that leads to corneal stiffening with age [12].

Biological scaffolds derived from the ECM of tissues have been used as scaffolds for tissue engineering and wound care products [13,14]. To engineer these materials, human and animal tissues are processed to remove as much cellular debris as possible to isolate the ECM. After treatment, they can be formed into sheets, gels, or particles, and if the tissues are carefully processed to remove immune-genic components, ECMs derived from animals can be successfully used in human clinical applications. For example, some wound care products used clinically today are derived from porcine bladder and small intestine [15]. In the case of ophthalmic applications, tissue-derived material such as amniotic membrane is employed for treating ocular wounds and limbal stem cell delivery [16,17]. However, applying decellularized ECM materials to modulate wound healing in the eye has not been explored yet. Beyond mere structural support, these materials contain biological cues that regulate cell activities such as proliferation, migration and differentiation of multiple cell types including stem cells and immune cells [18,19], and these biological cues would be benefit corneal biomechanics and integrity.

In this work, we investigate the potential of tissue-derived biological particles (TDPs) in restoring cornea physical and biological properties in an enzyme-mediated ex vivo corneal ectatic model. Exposure to the tissue particles increased cornea mechanical properties, restored matrix architecture and decreased inflammatory gene expression. These results open the door to the potential therapeutic use of tissue-derived particles in cornea disease.

## 2. Materials and Methods

### 2.1. Preparation of TDP-Suspended Medium

Porcine eye globes, knee joints and lymph nodes were purchased from local slaughter house. Corneas (COR) from the eyes, cartilage (CART) from the knee joints, and lymph node (LN) tissue were dissected from adjoining tissue. The tissues were thoroughly washed with tap water and frozen before processing. Using a standard decellularization process [20], the tissues were thawed and ground with knife mill, followed by treatment with 3% peracetic acid (Sigma Aldrich, St. Louis MO, USA) on a stir plate for 4 h (500 mL 3% peracetic acid per 20 g of minced tissue). After acid treatment, the minced tissue was washed extensively with tap water followed by deionized (DI) water until the pH was neutralized. The samples were then treated with 5% Triton-X (Sigma Aldrich, St. Louis, MO, USA) for 48 h, followed by a final digestion with DNase (Roche) overnight. The processed tissue was lyophilized and then cryomilled into microparticles before use. To prepare TDP-suspended medium, the TDP microparticles were suspended in full medium, which contained DMEM (Gibco) +10% Fetal bovine serum (FBS) (Gibco) +1% antibiotic-antimycotic (Gibco) at concentration of 1 mg/mL overnight, and then filtered through a 0.22 μm filter before use.

### 2.2. Ectatic Corneal (KC) Culture in TDP-Suspended Medium

Fresh albino rabbit eyes were purchased from Pelfreez Biologicals (Rogers, AR, USA). The ectatic model was previous developed in our lab [21], briefly, to create the ex vivo keratoconus-like model (KC) corneas, the eyes were treated with 0.1 U/mL chondroitinase ABC (C36675U Sigma Aldrich, St. Louis, MO, USA) by immersing the anterior surface in enzyme solution for 2 h. Following enzyme treatment, the eye globes were washed with full medium several times. The KC corneas were then treated to the different TDPs by submersing the anterior segment in TDP-suspended medium containing 4% dextran-500 (Sigma Aldrich, St. Louis, MO, USA) for 18 h. The dextran was added to reduce extensive swelling of the ex vivo corneas. Control corneas without enzyme treatment were cultured in 4% dextran full medium without TDP microparticles.

### 2.3. Optical Coherent Tomography (OCT)

The ex vivo eye globes right after treatment were imaged with Envisu R4110 SDOCT system (Bioptigen, Morrisville, NC, USA) with light source with an output power of 10 mW, and an effective bandwidth of 105 nm centered at 845 nm. The transversal resolution is approximately 12 μm. The scanning area of each cornea had a width of 12 mm and depth of 1.2 mm.

### 2.4. Transmission Electron Microscopy

Samples before and after TDP treatment were stored in 20% dextran-500 (Sigma Aldrich, St. Louis, MO, USA) phosphate buffered saline (PBS) (Gibco) overnight to minimize any swelling effects before fixation. The fixative contained 3% paraformaldehyde, 1.5% glutaraldehyde, 5 mM MgCl_2_, 5 mM CaCl_2_, 2.5% sucrose, and 0.1% tannic acid in 0.1 M sodium cacodylate buffer, at pH 7.2. Samples were fixed overnight at 4 °C, then post-fixed in 1% osmium tetroxide, followed by staining with 2% aqueous uranyl acetate and dehydrated in increasing concentrations of ethanol (from 30% to 100%). Thin sections (60–90 nm) were cut and stained with lead and uranyl acetate. Sections were observed with Philips/FEI BioTwin CM120 Transmission Electron Microscope at 80 kV.

### 2.5. Image Analysis

TEM images were analyzed using MATLAB program, using code that was previously established [9]. Original images were binarized using an adaptive thresholding method. To estimate the density of fibers, a window of fixed size (300 × 300 dpi) was randomly localized. Within the window, the number of fibers per unit area was measured and recorded. Two hundred windows were measured independently in one image and at least 3 images were measured. The density of fibers was estimated using the median number.

### 2.6. Tensile Test

Uniaxial tensile testing was carried out to determine the stress-strain behavior of the corneas before and after TDP treatment. Samples right after treatment (*n* = 6 in each group) were cut into 5 mm stripes, and the thickness of the stripes was recorded. Superglue was used to fix both ends of the corneas to sample clamps to avoid slippage. The tensile test was performed at the rate of 0.1 mm/s on Bose EnduraTEC ELF3200 using a 250 g load cell. Load and displacement were recorded by software WinTest.

### 2.7. Differential Scanning Calorimetry (DSC)

The thermal stability of the corneas was assessed using the DSC 8000 (Perkin Elmer, Waltham MA, USA). Samples right after treatment (*n* = 3 in each group) were obtained using a 6 mm biopsy punch and placed in 30 μL aluminum pans and crimp-sealed. An empty pan was used as reference in all samples. DSC was performed over a temperature range of 10 °C to 95 °C, at a rate of 5 °C per minute under a 20 mL/min nitrogen flow. Denaturation temperatures were detected and analyzed with the Pyris software (Perkin Elmer) version 10.1.

### 2.8. Gene Expression

Corneas right after treatment (*n* = 6 in each group) were flash-frozen in liquid nitrogen and broken into small segments using ceramic pestles. The corneal segments were treated with Trizol (ThermoFisher, Waltham, MA, USA) and the total RNA was collected with RNeasy Mini Kit (Qiagen). The mRNA concentrations were quantified by Nanodrop-1000 spectrophotometer (NanoDrop Technologies, Wilmington, DE, USA), and then reverse-transcribed with High Capacity cDNA kit (Applied Biosystems). Quantitative polymerase chain reaction (qPCR) was performed with SYBR Green qPCR Mastermix (Applied Biosystems), and expression levels were normalized using housekeeping gene Glyceraldehyde 3-phosphate dehydrogenase (Gapdh). The primers of the targeted genes are listed in Table 1.

### 2.9. Statistical Analysis

Student’s t-test was used to statistically examine differences between control, KC and TDP treated corneas in terms of collagen fibril diameter and density. Young’s modulus, and gene expression were analyzed with Prism 6.01 (GraphPad Software, Inc., San Diego, CA, USA) using one-way analysis of various (ANOVA). All experiments were repeated twice independently, with *n* = 6 for each experimental group. *p*-values < 0.05 were considered statistically significant.

## 3. Results

**Figure 1:**
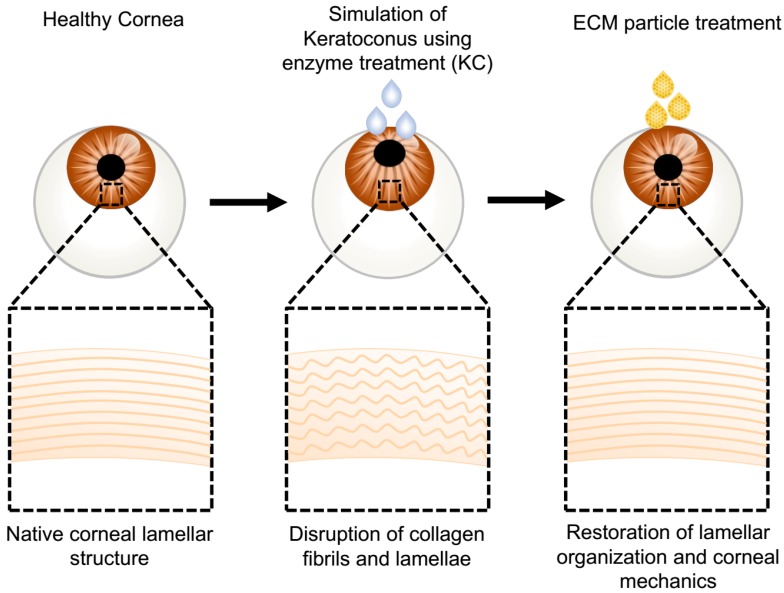
Schematic of treatment procedure to produce the ex vivo ectatic models and subsequent tissue-derived biological particles (TDP) treatments. Fresh rabbit eye globes were treated with chondroitinase to create an ex vivo corneal ectatic keratoconus model (KC) that simulates the ultrastructural damage observed in keratoconus corneas. The modeled KC corneas were cultured in TDP-suspended medium. Optical, mechanical, and ultrastructural characterization demonstrated the restoration of the ultrastructure and mechanical integrity of the KC corneas following TDP treatment.

To determine the potential use of processed TDPs as an alternative to photo-crosslinking, we first enzymatically induced corneal weakening and degradation to simulate corneal ectasia. Then, the ex vivo keratoconus-like model (KC) globes were exposed to various TDP-suspended medium (Figure 1). Changes in the cornea mechanical and thermal properties, matrix ultrastructural organization, and gene expression were compared.

### 3.1. TDP Treatment Restored Corneal Fibril Density

**Figure 2:**
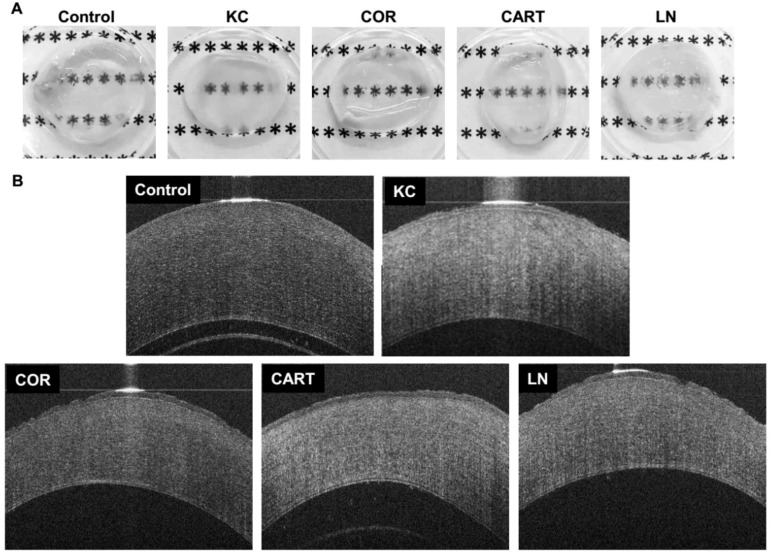
Gross observation and cornea Optical coherent tomography (OCT) before and after TDP treatment. (**A**) gross pictures of corneas with different treatments dissected immediately following tissue culture, and (**B**) OCT images of control corneas, corneas with chondroitinase digestion (KC) and corneas (COR) from the eyes, cartilage (CART), and lymph node (LN) TDP treatments.

Gross pictures of the corneas dissected immediately after the tissue culture demonstrated qualitative optical property changes after enzymatic digestion and TDP treatment (Figure 2A). Chondroitinase treatment resulted in decreased transparency of the modeled KC corneas compared to the control corneas. Cornea tissue derived particles (COR) treated corneas restored transparency grossly to a level that was comparable to control corneas. Cartilage (CART) and lymph node (LN) treated corneas also increased transparency compared to KC corneas.

Rabbit corneas are prone to swelling immediately post-enucleation, despite intact epithelial and endothelial cell layers. The swelling is exacerbated if any damage to the collagen structure occurs. Optical coherent tomography (OCT) imaging provides further visual characterization of the corneal changes with degradation and TDP treatment (Figure 2B). Control corneas (not treated with degradative enzymes) cultured in full medium (without TDP) for 18 h were approximately 500–600 µm thick. KC Corneas after enzymatic digestion, resulted in explants with a thickness comparable to control corneas and hyper-reflection of light in the OCT images, despite the lower collagen and proteoglycan contents. This is indicative of extensive swelling and corresponds to the reduced transparency after KC treatment in the gross images. KC corneas which were treated with TDP, however, did not swell and were approximately 400 µm in thickness. TDP treated corneas also did not demonstrate the hyper-reflection observed in the KC corneas. The light reflection in the OCT images of the COR corneas was markedly similar to normal control corneas.

**Figure 3:**
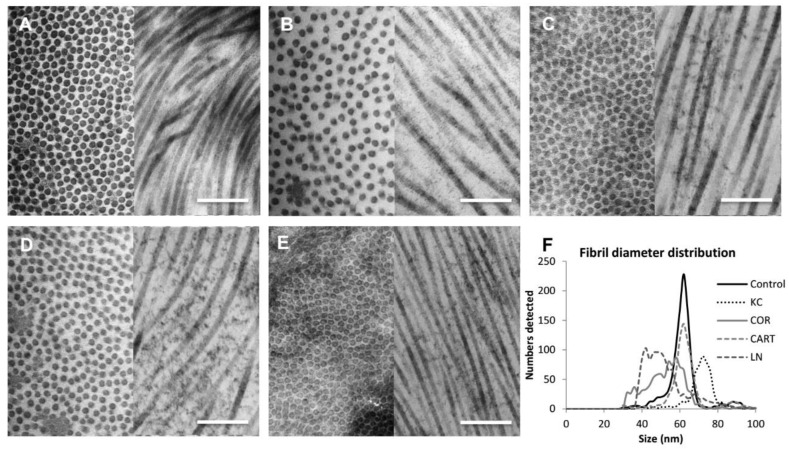
Characterization of corneal ultrastructure and fibril distribution. TEM images revealed ultrastructural changes of the corneas before and after treatments. (**A**) Control corneas (**B**) KC corneas presented thicker fibril diameters and lower fibril density as compared to the control corneas. (**C**) Corneal TDP treated corneas (**D**) Cartilage TDP treated corneas, (**E**) Lymph node TDP treated corneas, (**F**) Fibril diameter distribution among control, KC and three TDP groups. Scale bar = 1 µm.

The cornea extracellular matrix ultrastructure significantly changed with TDP treatment (Figure 3). Chondroitinase enzyme activity degraded proteoglycans in the cornea, which led to a weakened inter-and intra-collagen molecule binding and resulted in a lower fibril density and a changed fibril diameter compared to normal controls (Figure 3A,B). The loss of proteoglycan content also contributed to a larger average fibril diameter, likely due to loosened intra-fibrillar interactions within the fibril (Figure 3F). KC corneas had a larger average fibril diameter and lower fibril density albeit with a similar pattern of fibril distribution compared to normal control corneas. Treatment of the KC corneas with TDPs significantly increased fibril density, and the horizontal view of the collagen arrangement showed extra staining of proteoglycans (Figure 3C,E). However, collagen fibril diameter and density differed depending on the type of TDP treatments (COR, CART, and LN). In comparison to normal controls, the COR exposure produced smaller median fibril diameter sizes but a larger fibril diameter distribution (Figure 3C). Culture with CART produced a similar fibril diameter (Figure 3D) while LN particles produced the smallest fibril diameters (Figure 3E). Additionally, the LN exposure stimulated the highest collagen fibril density.

### 3.2. TDP Treatment Enhanced Corneal Tensile Strength And Thermal Stability

**Figure 4:**
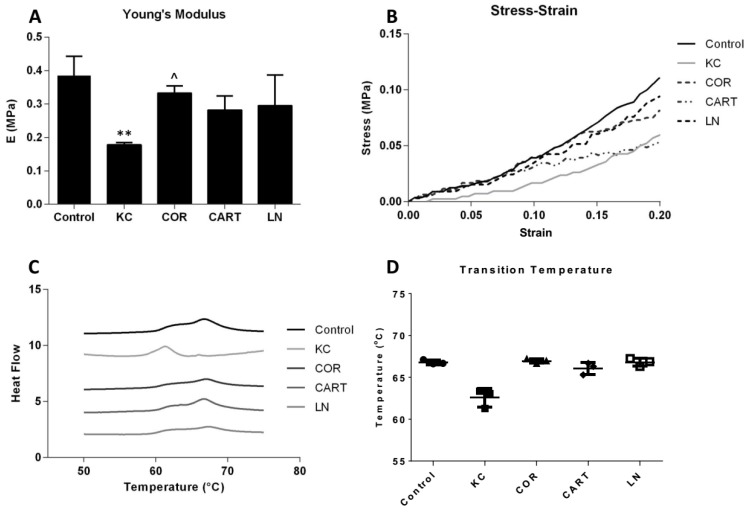
Mechanical and thermal stability before and after TDP treatments. (**A**) Young’s modulus and (**B**) stress-strain curves of control, KC and TDP treated corneas. *n* = 6 in each group, * refers to *p* < 0.05 compared to control corneas, and ^ refers to *p* < 0.05 compared to KC corneas. COR treatment significantly increased Young’s modulus compared to the KC corneas. CART and LN treatment presented reinforcement effects but not statistically significant. (**C**) Schematic DSC curve and (**D**) transition temperature of control, KC and TDP treated corneas. All TDP treatments restored thermal stability of the corneas. *n* = 3 in each group.

TDP treatment enhanced corneal mechanical properties and extracellular matrix thermal stability. Uniaxial tensile testing demonstrated a significant reduction in the elastic modulus of KC corneas after enzyme treatment. Following TDP treatment, especially the COR group, the elastic modulus of the KC corneas was restored and there was no statistical difference between the moduli of COR treated corneas and the normal controls (Figure 4A). CART and LN treatment also reinforced the matrix, but the changes were not statistically significant. Representative stress-strain curves also demonstrate the effect of the reinforcement through TDP treatment at higher strain values (Figure 4B). DSC thermograms, a measure of the matrix organization and stability, display variations in the denaturation temperatures between the control and KC corneas via broad peaks over the 60–70 °C range (Figure 4C). While KC corneas had significantly lower mean denaturation temperatures (62.57 ± 1.11 °C) as compared to the control corneas (66.8 ± 0.27 °C), the COR, CART and LN TDP treatment restored thermal denaturation temperatures in all three groups to 66.96 ± 0.28 °C, 66.08±0.71 °C, and 66.80 ± 0.47 °C, respectively (Figure 4D).

### 3.3. TDP Treatments Maintain Keratocytes Phenotype

**Figure 5:**
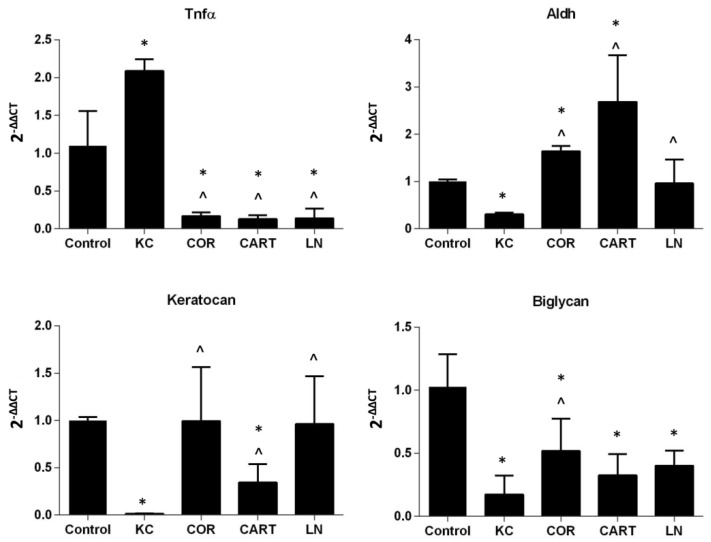
Gene expression of keratocytes after TDP treatment. Pro-inflammatory marker *Tnfα*, keratocyte markers *Aldh*, *keratocan* and *Biglycan* were evaluated in normal, KC and TDP treated corneas. *n* = 6 in each group, * refers to *p* < 0.05 compared to normal corneas, and ^ refers to *p* < 0.05 compared to KC corneas.

TDP treatment reduced pro-inflammatory gene, *Tnfa*, expression, and supported the maintenance of keratocyte marker gene expressions (Figure 5). Enzymatic digestion significantly upregulated expression of *Tnfa* in KC corneas. After the digested KC corneas were treated with TDP containing medium, *Tnfα* expression significantly decreased to levels lower than the control group. In parallel with the increase in *Tnfα* expression, KC corneas down-regulated expression of the keratocyte markers *keratocan*, *Aldh*, and *biglycan*. Following treatment of KC corneas with TDP-suspended medium, keratocyte marker expression was restored.

## 4. Discussion

Biological scaffolds used clinically today are derived from human or animal tissue sources. Matristem^®^, processed from porcine urinary bladder, is available in sheets or particles depending on the application and is approved for wound healing indications including partial or full thickness wounds, ulcers, and burns. Multiple studies highlight the clinical efficacy of the biological material in promoting wound healing [22,23]. OASIS^®^, another clinically-approved product, is manufactured from porcine small intestinal submucosa. This matrix is also approved for wound healing applications, including treatment of chronic leg ulcers [24]. In both cases, the tissue-derived scaffold treatments accelerated wound closure compared to standard treatments. Biological materials have also been applied to ocular applications, specifically amniotic membrane. It is frequently used for corneal damage repair where it is purported to reduce inflammation and promote re-epithelialization of the ocular surface. A recent clinical study reported 80.7% of the patients with corneal ulcers or pterygium who received amniotic membranes as ocular grafts achieved full re-epithelialization with resolution of inflammation [25]. This proof of concept for using tissue-derived biological materials in ocular applications is supported by both the amniotic membrane results in the cornea and extra-ocular applications of other tissue products. In this study, we explored the application of tissue-derived materials isolated from 3 different tissues for a new ocular application, to reinforce corneal biomechanics.

Corneal ectatic disorders such as keratoconus often involve progressive degradation of collagen. Collagen degradation in turn severely weakens and deforms afflicted corneas, resulting decreased visual acuity. Disease pathogenesis is associated with inflammation in the cornea in addition to degradation of the cornea ECM. In a previously reported study [21,26], we established an ex vivo KC model using enucleated rabbit eye globes exposed to the degradative enzymes, in order to simulate several physical characteristics of KC corneas, such as decreased thermal and mechanical properties, a disrupted ultrastructure, as well as altered gene expressions. In the current study, we used our previously developed method to model KC and test the efficacy of TDP treatment. Chondroitinase ABC targets the glycosaminoglycan chondroitin sulfate (CS), thereby weakening the collagen-proteoglycan network of the cornea stroma, leads to the decrease in thermal stability and mechanics, as well as disrupted collagen alignment. Upon applying TDPs to the ectatic corneal models, the TDPs efficiently restored thermal and mechanical properties, and modulated the gene expression. We hypothesized that their application in KC may provide a source of biological materials to structurally reinforce the damaged tissue and, based on their wound healing properties, may reduce disease-associated inflammation.

The biomaterials used in this study were derived from cornea, cartilage, and lymph node tissues. Cornea and cartilage tissues are often regarded “immune privileged” due to the lack of vasculature and low cell numbers. They have a dense extracellular composed of collagen and tissue-specific proteoglycans. Proteoglycans in the cornea (decorin, keratocan, and lumican) [27] and cartilage (aggrecan, biglycan, and decorin) [28] play important roles in regulating collagen fibril diameter and interfibrillar crosslinking during development [9]. In contrast, lymph nodes are highly cellular structures thus when they are processed they consist of a number of plasma proteins. In addition, lymph nodes are abundant in lumican, a proteoglycan also relevant in the cornea [29]. The presence of the cornea-related proteoglycans in addition to collagens provides a potential source for tissue reinforcement in the weakened KC ECM. The corneal ultrastructure changed in response to TDP treatment and the type of response depended on the source of the tissue ECM (COR, CAR, and LN). This suggests that the unique composition of the ECM particles based on tissue source influenced KC ECM structural reinforcement. For example, lumican is known to regulate, and limit collagen fibril diameter growth during native corneal development [30]. Treatment with lumican abundant TDP (COR and LN) decreased average collagen diameters in the KC corneas. Correspondingly, LN treatment resulted in collagen fibers with the smallest fibril diameters, potentially due to a higher lumican content compared to COR. While the collagen diameters differed, all TDP treatments resulted in a highly packed collagen fiber arrangement, indicating formation of interfibrillar collagen crosslinks. The reduction of corneal thickness after TDP treatment as compared to the KC model also indicates the formation of inter- and intra-fibrillar crosslinking between collagen fibers contributes to the enhance biomechanics rather than an increased in ECM content. The ECM structure however may also be impacted by the inflammatory state of the resident cells and their matrix production versus secretion of degrading enzymes. The exact mechanism of how TDP particles initiated the collagen crosslinking will be further explored in detail by validating the collagen and proteoglycan sources that may be incorporated during the collagen crosslinking.

Keratoconus is associated with a pro-inflammatory state. Several pro-inflammatory markers such as *Interleukin-6* (*Il-6*) and *Tnfα*, along with *matrix metalloproteinase 9* (*Mmp9*) are elevated in the tear fluid of keratoconus patients [31]. Inflammatory genes were also found to be expressed by the corneal epithelium and stromal keratocytes at the corneal cone apex of keratoconus patients [32]. Moreover, a marked reduction in key cornea enzymes, such as *Aldh*, and proteoglycans such as *keratocan* was found in cultured keratoconic stromal cells [33,34]. Thus, a pro-inflammatory environment, and changes in keratocyte phenotype, both contribute to cornea stroma degeneration in keratoconus patients. In our previous study we used porcine-derived TDP treatment to reduce corneal scarring after trauma in a rabbit model, and we found TDP treatment minimize the corneal haze formation by downregulating pro-inflammatory gene expressions in the cornea such as *Tnfα*, *Mmp9*, and *iNOS* [35] without triggering any xenogeneic inflammatory response. We also found ECM microparticles presented anti-inflammatory potential in in vitro macrophages and corneal fibroblast culture and different in vivo wound models, including volumetric muscle loss model and rabbit corneal stromal wound model [35,36]. Similar to these observations in the literature, we found an increase in *Tnfα* gene expression and a significantly decreased expression of *keratocan*, *biglycan*, and *Aldh* in the KC model corneas.

KC model corneas treated with TDP demonstrated a response similar to one of today’s current clinical treatments for KC, cornea crosslinking. TDP corneal crosslinking reinforces corneal biomechanics while also reducing inflammation and promoting healthy keratocyte phenotype. Tissue-derived biological materials are a complex system that contain various biological cues, therefore further studies to investigate the key proteins, such as collagen, or proteoglycans that play critical roles in corneal reinforcement will be continued. As an example of this, proteomics and western blotting will be further investigated in an ex vivo and in vivo model [37,38]. These materials will also be validated in vivo as in a long-term study to validate the impact to local keratocytes at a protein production level, as well as investigate the most effective and practical delivery methods of these tissue-derived particles to the cornea.

## Figures and Tables

**Table 1:** Gene primers used to assess gene expression in TDPs treated corneas.

*Gapdh*	Forward	5′-CTCTGGAGTGGATGTT-3′
	Reverse	5′-CCATGGGTGGAATCATACTG-3′
*Tnfα*	Forward	5′-GTAGTAGCAAACCCGCAAGT-3′
	Reverse	5′-GGTTGTCCGTGAGCTTCAT-3′
*Keratocan*	Forward	5′-AGTACCAACAAGCTTCAGCC-3′
	Reverse	5′-ACCCAGATGACGAAACATATT-3′
*Aldh*	Forward	5′-GACGATAACTGCAGAGCACG-3′
	Reverse	5′-ACTCATTCGACAAGCAGACAG-3′
*Biglycan*	Forward	5′-GCATCCCCAAAGATCTGCC-3′
	Reverse	5′-CAACTTGGAGTATCGGAGCAG-3′
